# BM-MSCs differentiated to chondrocytes for treatment of full-thickness cartilage defect of the knee

**DOI:** 10.1186/s13018-020-01852-x

**Published:** 2020-10-06

**Authors:** Rodrigo Mardones, Alessio Giai Via, Gennaro Pipino, Claudio M. Jofre, Sara Muñoz, Edgar Narvaez, Nicola Maffulli

**Affiliations:** 1 CDMA, Santiago de Chile, Chile; 2 Department of Orthopaedic Surgery and Traumatology, San Camillo-Fortalini Hospital, Rome, Italy; 3 UCM Malta, Campus of Lugano, Lugano, Switzerland; 4 Department of Orthopaedic Surgery and Traumatology, Villa Regina Hospital, Bologna, Italy; 5 Department of Orthopedics and Physiotherapy, UCM University, Msida, Malta; 6 Regenerative Cell Therapy Center, Clinica Las Condes, Santiago, Chile; 7 Department of Radiology, Clinica Las Condes, Lo Fontecilla 441, Las Condes, Santiago de Chile, Chile; 8 Institute of Science and Technology in Medicine, Keele University School of Medicine, Thornburrow Drive, Stoke on Trent, England; 9 Department of Orthopedics and Physiotherapy, UCM University, Msida, Malta

**Keywords:** Mesenchymal stem cells, Cartilage injury, Articular cartilage, Knee, Collagen scaffold

## Abstract

**Background:**

Full-thickness articular cartilage injury of the knee is a major cause of disability. The aim of this study is to assess the outcome of patients treated with differentiated to chondrocytes bone marrow mesenchymal stem cells (BM-MSCs) cultured on a collagen type I/III (Chondro-Gide®) scaffold. The secondary aim was to confirm the absence of adverse events.

**Methods:**

Fifteen patients (19 knees) with symptomatic full-thickness cartilage defects of the knee were enrolled. Bone marrow was harvested from the iliac crest, BM-MSCs were prepared, and expanded cells were grown in a standard medium or in a standard culture medium containing TGF-β. BM-MSCs differentiated to chondrocytes were seeded in a porcine collagen type I/III scaffold (Chondro-Gide®) and cultured in TGF-β containing media. After 4 weeks, the membrane was sutured on the cartilage defect. All patients underwent plain radiographs (antero-posterior, lateral, and axial view of the patella) and MRI of the affected knee. The Oxford knee score, the Lyhsolm scale, and the VAS score were administered to all patients. At final follow-up a MRI for the study of articular cartilage was undertaken.

**Results:**

The mean size of the cartilage lesions was 20 × 17 mm (range, 15 × 10 mm–30 × 30 mm). At final follow-up, the median Oxford knee score and Lyhsolm scale scores significantly improved from 29 (range 12–39; SD 7.39) to 45 (range 24–48; SD 5.6) and from 55.5 (range 25–81; SD 17.7) to 94.5 (58–100; SD 10.8), respectively. Pain, according to the VAS score, significantly improved. Sixty percent of patients reported their satisfaction as excellent, 20% as good, 14% as fair, and 1 patient as poor.

**Conclusion:**

The treatment of full-thickness chondral injuries of the knee with differentiated to chondrocytes BM-MSCs and Chondro-Gide® scaffold showed encouraging outcomes. Further studies involving more patients, and with longer follow-up, are required to evaluate the effectiveness of the treatment and the long-term results.

## Introduction

Chondral injuries of the knee are a major cause of short- and long-term disability, and their management is challenging. Articular cartilage, a highly organized tissue with considerable durability, has limited intrinsic healing capability [1]. Cartilage injuries frequently result in knee pain, functional impairment and early osteoarthrosis. Currently, the treatment for end-stage degenerative knee joint pathology is arthroplasty. Surgery for symptomatic cartilage defects, including microfractures, osteotomies, or autologous osteochondral graft transplantation, aims to restore joint congruity and minimize further deterioration [2]. However, the long-term results are often poor; therefore, great interest arose on the development of regenerative medicine and tissue engineering approaches [3–5]. Recently investigations have reported on the local implantation or intra-articular injection of concentrated bone marrow or adipose-derived MSCs, but the results are controversial [6, 7].

We report the results of a novel treatment of full-thickness cartilage defects of the knee. Bone marrow mesenchymal stem cells (BM-MSCs) were harvested from the iliac crest, expanded in a laboratory facility, differentiated to chondrocytes on a collagen-based scaffold (Chondro-Gide®), and finally the scaffold was sutured on the cartilage defect of the knee. The primary aim of this study was to assess the mid-term results of such procedure. The secondary aim was to confirm the absence of adverse events. The working hypothesis is that the implantation of BM-MSCs differentiated to chondrocytes with a collagen scaffold significantly improved pain and function of patients with full-thickness cartilage defects of the knee.

## Materials and methods

We retrospectively analyzed the prospectively collected data of 15 patients who underwent surgery for articular cartilage defect of the knee between June 2012 and January 2015. All patients had signed a written consent, and the study was approved by the local Internal Review Board (IRB-Clinica Las Condes, Santiago de Chile, Chile).

### Inclusion and exclusion criteria

The inclusion criteria were patients with symptomatic full-thickness cartilage defect of the knee despite a minimum of 3 months of conservative treatments, a Kellgren-Lawrence OA grade 1 or 2, age older than 18 years. The exclusion criteria were patients older than 50 years, knee instability, varus or valgus malalignment higher than 5°, and previous intra-articular fractures. Patients with rheumatological conditions, chronic renal failure, previous joint infections, neoplasia, or psychiatric disorders were excluded. Patients who were not able to sign the informed consent or to follow the post-operatory instructions were not included into the study. Fifteen patients met the inclusion criteria.

### Patient assessment

A detailed physical examination was conducted in all patients, who were examined by fully trained orthopedic surgeons with a special interest in knee surgery. All patients underwent standard weight-bearing anterior–posterior (AP) plain radiographs, lateral views of the knee, and axial view of the patella. An MRI confirmed the focal cartilage defect. Osteoarthritis of the knee was evaluated according Kellgren-Lawrence osteoarthrosis classification [8]. The grade of the cartilage defect was classified according the Outerbridge classification. The Oxford knee score [9] and the Tegner-Lyhsolm scoring scale [10] were administered to all patients before surgery (T0) and final follow-up (T1). According the Oxford knee score, scores lower than 19 were considered as poor outcomes, from 20 to 29 as fair, from 30 to 39 as good, and score equal or higher than 40 as excellent. Scores lower than 65 were considered as poor according the Tegner-Lyhsolm scoring scale, from 65 to 83 as fair, from 84 to 90 as good, and scores higher than 90 as excellent. The VAS score was also administered preoperatively and at each control. At final follow-up, an MRI was also performed. All patients were examined by a fellow who had not been involved in their original management.

### Isolation, expansion, and characterization of BM-MSCs

BM-MSCs were cultured from each of the 15 patients reported in the present investigation. Briefly, 40 mL of bone marrow (BM) were aspirated from the iliac crest, and mononuclear cells were isolated from BM aspirates by density gradient centrifugation using LymphoprepTM (Stemcell Technologies, Cambridge, MA, USA). The BM mononuclear cells were then plated at a density of 3.5 × 10^5^ cells/cm^2^ in T225 flask with 40 mL of customized alpha modified Eagle medium (α-MEM) (Gibco, Invitrogen, USA) supplemented with 10% (v/v) Australian fetal bovine serum (FBS) (Corning, NY, USA) and 1% (v/v) penicillin-streptomycin (50 μg/mL) + amphotericin B (Pen-strep, Biological Industries, USA) and incubated at 37 °C in humidified atmosphere containing 5% CO_2_ [11]. After 48 h, the non-adherent cells were washed off gently with Dulbecco’s phosphate-buffered saline (DPBS; Hyclone, USA), and the flasks were microscopically verified for fibroblast-like adherent cells. After 14 to 21 days, cells were trypsinized using 0.25% trypsin-EDTA (Biological Industries, USA) and passaged at 10,000 cells/cm^2^ for another 7 to 12 days, followed by a final cell harvesting and counting to determine the final cell number before seeding them into the scaffold. At each step of harvesting, population doubling level (PDL) was calculated as followed: PDL = *X* + 3.322 (log *Y*–log *I*), where *X* = initial population doubling level, *I* = initial cell number seeded into the vessel, and *Y* = final cell yield, or the number of cells at the end of the growth period (Table 1). For cell surface antigen analysis, cells were harvested, washed with cytometer buffer (PBS + 0.2% BSA + 0.01% sodium azide (all from Sigma-Aldrich), and incubated with the specific labeled antibodies in cytometer buffer for 20 min at 4 °C. Antibodies for human cell surface antigens CD11b-AF488, CD29-PE, CD73-PE, CD90-FITC, CD105-PE, CD34-PE, CD19-PE, CD45-FITC, and HLADR-PE were purchased from R&D Systems (Minneapolis, MN, USA). In all experiments, matching isotype antibodies were used as negative controls. Data were collected using a fluorescence-activated cell sorting (FACS)-Vantage-SE flow cytometry system running CellQuest software (BD). The fluorescence signals were collected using logarithmic amplification (BD Biosciences) and analyzed on FlowJo analysis software (FlowJo LLC, Ashland, OR, USA).

**Table 1:** Characteristics of the patients. The grades of the defect have been classified according the Outerbridge classification

Patients	Age	BMI	Follow-up (months)	Grade of the injury	Site of the defect	Width of the defect (mm)	Previous surgery
n.1	46	25.8	46	IV III	Medial condyle Tibial plateau	30 × 18 20 × 20	No
n.2	37	32.4	45	IV IV	Patella Troclea	35 × 30 35 × 30	Knee arthroscopy and microfractures
n.3	25	28.3	46	IV	Patella	12 × 14	Microfractures and extensor apparatus realignment
n.4	33	21	46	IV	Patella	15 × 15	No
n.5	33	24.8	36	III	Medial condyle	26 × 30	Medial meniscectomy and meniscal transplant
n.6	18	20.7	37	IV	Medial tibial plateau	15 × 10	Arhtroscopic partial meniscectomy
n.7	38	21.6	29	IV	Medial condyle	25 × 20	Knee arthroscopy and microfractures
n.8	34	27	30	IV	Patella Troclea	10 × 20 10 × 15	MPFL reconstruction
n.9	34	26.5	30	III	Medial condyle	20 × 20	ACL reconstruction
n.10	51	27.5	28	IV	Medial condyle	30 × 25	Knee arthroscopy, microfractures, partial meniscectomy
n.11	40	23	18	IV IV	Patella Troclea	30 × 20 20 × 20	Knee arthroscopy, microfractures
n.12	24	32.4	24	III	Lateral condyle	20 × 15	Open wedge distal femoral osteotomy
n.12	24	32.4	27	IV	Lateral condyle	20 × 20	Open wedge distal femoral osteotomy
n.13	48	26	24	IV	Medial and lateral Patella’s facets	30 × 9	No
n.14	20	26	24	IV	Patella	10 × 15	No
n.15	32	25.9	24	IV	Lateral condyle	20 × 18	No

*MPFL* medial patella-femoral ligament, *ACL* anterior cruciate ligament

#### In vitro chondrogenesis and cell seeding of BM-MSC into scaffold

BM-MSCs from the 15 patients were seeded at a density of 1 × 10^6^ cells/cm^2^ in 1 mL of standard culture medium per 4 × 5 cm piece of Chondro-Gide in non–tissue culture coated plates. After 30 min of incubation at 37 °C to allow the cells adhere to the cell scaffolds, an additional 9 mL of standard culture medium was added. This was repeated for 3 days according to our cell seeding protocol. From day 5, chondrogenic induction was started with chondrogenic induction medium containing α-MEM + ITS premix (Sigma-Aldrich), ascorbic acid (37.5 ug/mL) (Sigma-Aldrich B4461) and TGF-β1 10 ng/mL (B&D, NY, USA). Culture medium was replaced 3 times per week for 15 days. Histological analysis was performed to verify cell adhesion and glycosaminoglycans (GAG) deposition to the scaffold before surgery.

### Surgical technique

**Fig. 1:**
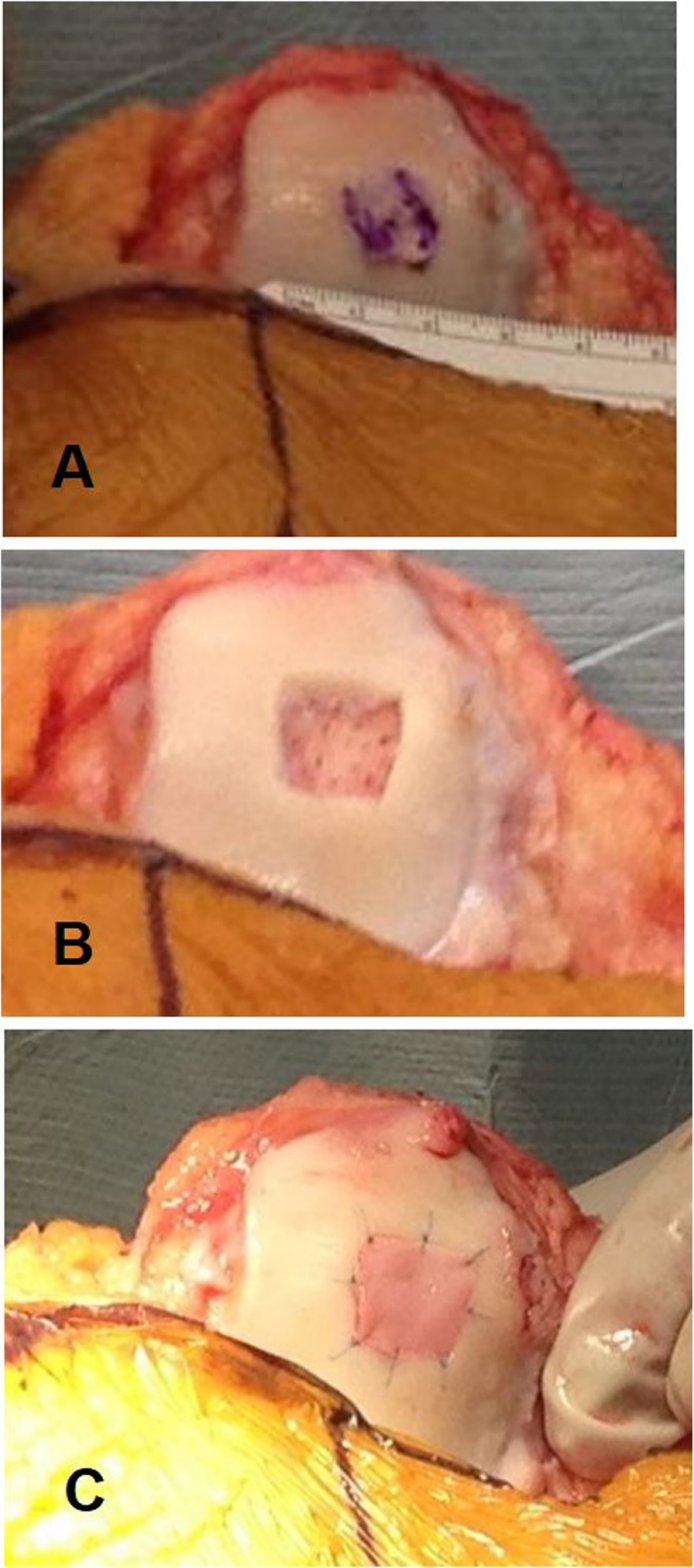
A 25-year-old active male patient with patellar maltracking and patellofemoral pain syndrome, who has received 4 knee procedures before the final cartilage repair. The patient reported pain and swelling because of a large grade IV chondral injury of the patella. **a** After a medial parapatellar arthrotomy, the patella was exposed and the cartilage defect identified. **b** The cartilage defect was prepared and microfractures have been performed. **c** The Chondro-Gide® scaffold containing BM-MSCs differentiated to chondrocytes was sutured with n.6.0 non-absorbable suture.

Under spinal anesthesia and with the patient supine, the lower limb was prepped and draped in the usual sterile fashion. A tourniquet was applied at the upper thigh, the lower limb exsanguinated, and the tourniquet inflated to 300 mmHg. A midline incision and a medial parapatellar arthrotomy were performed. The cartilage defect was identified, debrided, and the borders of the defect were regularized up to obtain a stable cartilage layer. The size of the defect was measured. After performing microfractures, the collagen matrix scaffold containing BM-MSCs differentiated to chondrocytes was sutured on the peripheral healthy cartilage using Prolene® (Ethicon) n.6.0 suture (Fig. 1). The tourniquet was deflated. The joint capsule was sutured. The wound was closed with subcuticular 2.0 Vicryl suture, and a sterile dressing was applied. All patients received intravenous controlled analgesia for 24–48 h after surgery, and standard thromboembolic prophylaxis with low molecular weight heparin (LMWE) for 30 days.

### Postoperative protocol

All patients received the same rehabilitation program. Patients were walking with two crutches without weight-bearing on the operated leg for 4 weeks. Passive motion of the knee was encouraged from the first post-operatory day, and a continuous passive motion device was indicated twice a day for 4 weeks, for at least 20 min and up to 1 h per each session. Isometric strengthening exercises of the quadriceps and gluteus muscles were performed. The patients were examined at 4 weeks after surgery, when progressive full weight-bearing was initiated. The patients begun a supervised walk training program, proprioceptive exercises, and continue strengthening exercises. Patients were encouraged to walk without crutches as tolerated from the 6th post-operative week.

### Statistical analysis

Descriptive statistics are presented as mean (±SD). Student’s *t* test for independent samples was used to detect for differences between baseline and follow-up for each variable. To assess reliability and variability of the measures, we calculated the coefficient of variation (CV = SD/mean %). A *p* value < 0.05 was considered statistically significant. Statistical analysis was performed by using SigmaPlot 11.0 software (Systat Software, Tulsa, OK, USA).

## Results

### MSC and collagen membrane characterization

**Fig. 2:**
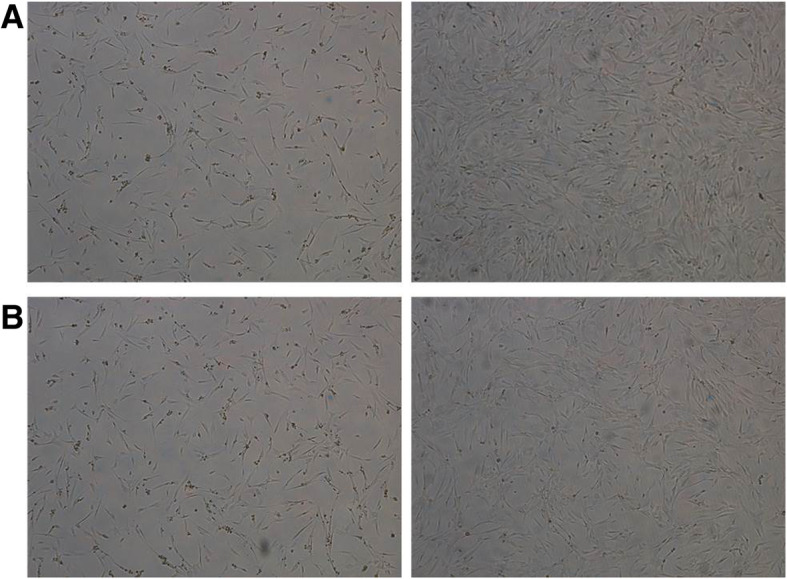
Representative images of cultured BM-MSCs in different states of confluence of two patients (**a**, **b**). On the left side, the confluence is about 30–40%, while on the right confluence is about 80%, prior to trypsinization for use in subsequent experiments

**Fig. 3:**
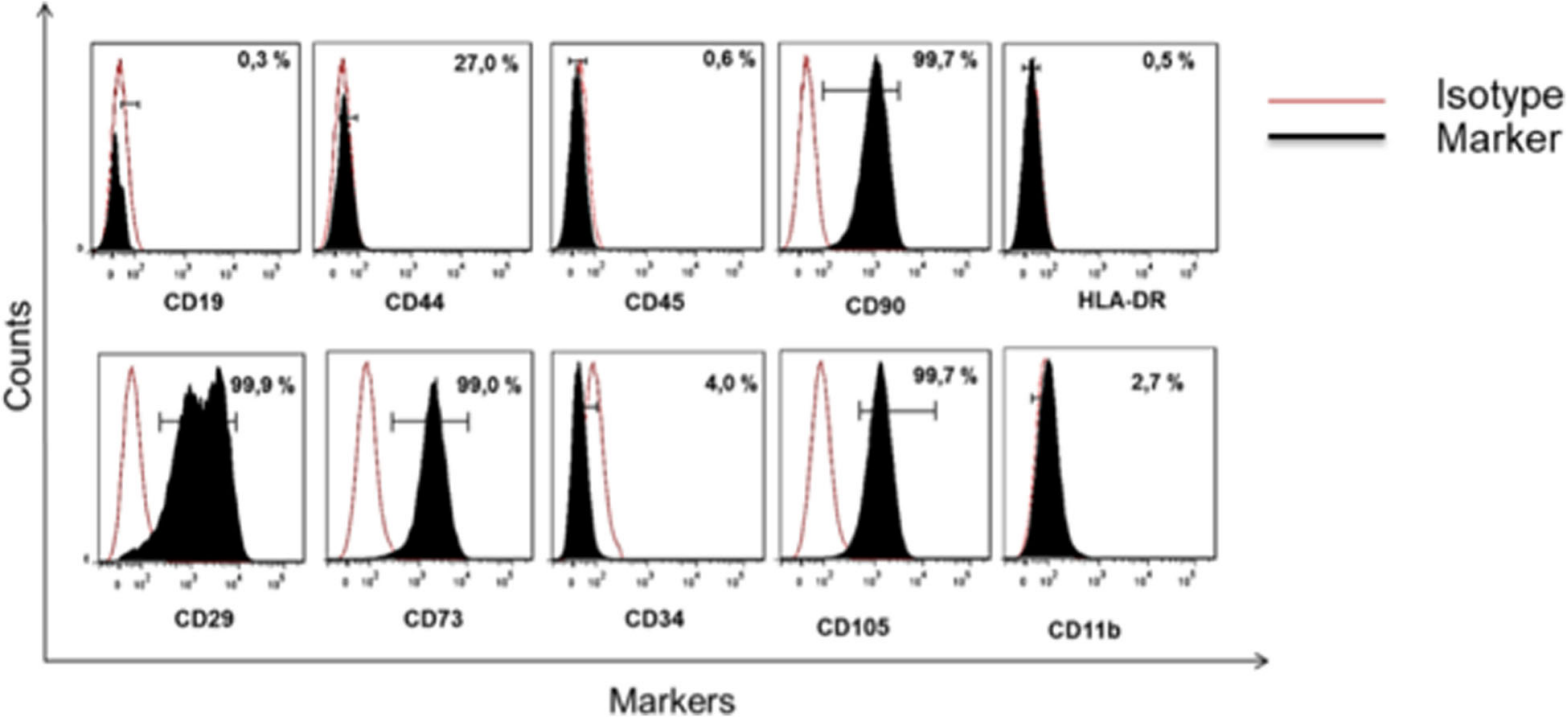
Representative histograms showing antigen expression in bone marrow-derived MSC. From left to right CD19, CD44, CD45, CD90, HLA-DR, CD29, CD73 CD105, CD73, CD34, CD105, and CD11b. Black-filled histogram: antigen expression; solid red line: auto-fluorescence control

Bone marrow aspirates with a mean volume of 42 ± 5 mL were collected from each of 15 patients. BM-MSCs were isolated by plastic adherence and expanded in tissue-culture flasks under GMPc culture conditions. Following cell isolation, BM-MSCs exhibited spindle fibroblast-like morphology in culture (Fig. 2), and they expressed classical MSCs surface markers, such as CD105, CD90, and CD73, in the absence of hematopoietic cell surface markers CD34 and CD45 (Fig. 3). In each of 15 patients, the above phenotype was consistent, confirming the MSCs phenotype previously described.

**Fig. 4:**
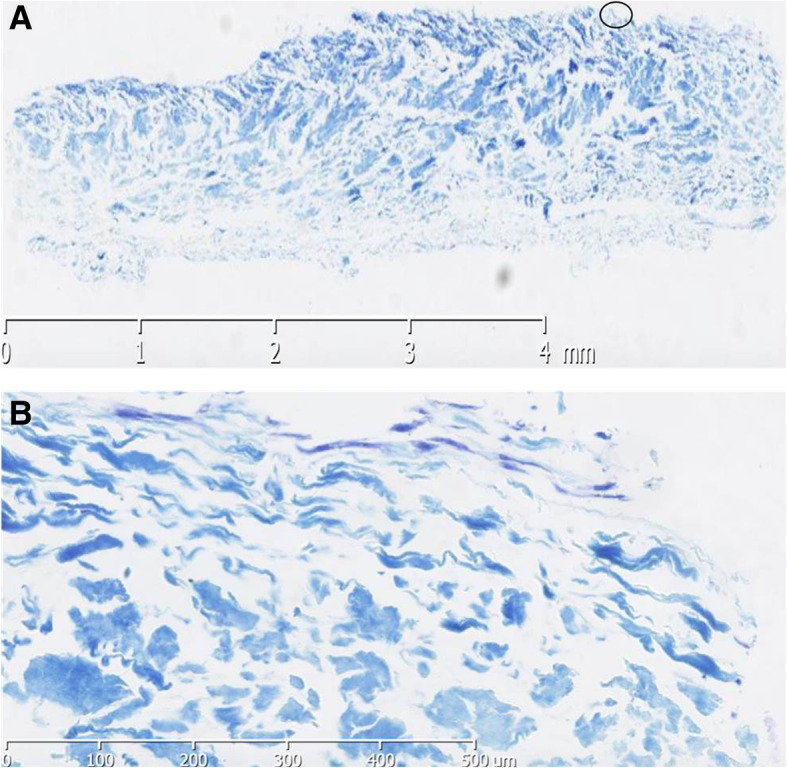
Histological analysis of BM-MSCs differentiated to chondrocytes in Chondro-Gide Scaffold. **a** Complete scaffold stained with 2% toluidine blue showing GAG deposition, indicating the presence of hyaline cartilage producing chondrocytes. **b** Section of collagen membrane showing cells adhered to the periphery of the scaffold. Circle indicates section in **a**

According to our scaffold seeding protocol, 1 × 10^6^ BM-MSCs with no more than P3 were seeded at day 1 on the porous side of the scaffold; the same quantity of cells was seeded on the smooth side of the scaffold on day 2, and on day 3, 1 × 10^6^ cells were seeded again on the porous side of the scaffold. From day 5 on, chondrogenic medium with α-MEM + ITS premix (Sigma-Aldrich BC6354), ascorbic acid (37.5 ug/mL) (Sigma-Aldrich B4461), and TGF-β1 10 ng/mL (B&D, NY) were added and changed every 2 days. On day 15, the scaffolds were harvested, fixed in 10% neutral buffered formalin for 24 h, then processed and paraffin embedded for histological analysis (Fig. 4). The cells showed a high rate conversion to chondrocytes, with a mean cell density attachment of 16% ± 3.6. The MSC differentiated to chondrocytes were more evident at the periphery of the scaffold with abundant GAG deposition on the complete scaffold, acknowledging that the MSC had differentiated to chondrocytes.

### Surgical findings and clinical outcomes

Four patients had a bilateral cartilage injury of the knee, for a total of 19 knees. The mean size of the cartilage lesions was 20 × 17 mm (range, 15 × 10 mm–30 × 30 mm). The characteristics of the patients and of the cartilage defects are shown in Table 1. The mean follow-up was 32 months (range, 12–46 months, SD 9 months). No patients were lost to follow-up.

Before treatment, the median Oxford knee score was 29 (mean 27.3; range 12–39; SD 7.38), and the median Tegner-Lyhsolm scoring scale was 55 (mean 55.9; range 25–81; SD 17.7). At final follow-up, the median Oxford knee score and Lyhsolm scale scores significantly improved to 45 (mean 43.3; range 28–48; SD 5.6) and 94.5 (mean 91.0; range 58–100; SD 10.8), respectively, (*p* < 0.01 for both). The VAS score showed a statistically significant improvement from its preoperative value to final follow-up. The median preoperatory VAS score was 4 at rest (mean VAS 3.8; range 2–6, SD 1.37) and 7 (mean VAS 6.25; range 3–8, SD 1.8) during working or sporting activities, while at final follow-up, it had improved to a median of 0 at rest (mean VAS 0.5; range 0–3, SD 1.09) and 1 during working or sporting activities (mean 1.6; range 0–5; SD 1.74) (*p* < 0.01). With regard to overall satisfaction with the surgery, 60% of patients reported their satisfaction as excellent, 20% as good, 14% as fair, and 1 patient as poor. Most patients returned to practice sports; in particular one patient returned to run marathons, and another one returned to run mountain marathons. The latter patient reported mild discomfort and some swelling after competition.

One patient developed stiffness of the operated knee and required manipulation under anesthesia and arthroscopic arthrolysis. No further complications were reported in this series, both in the short- and long-term. In particular, the MRI at the final appointment did not evidence hypertrophy of the graft in any patient.

### Imaging findings

**Fig. 5:**
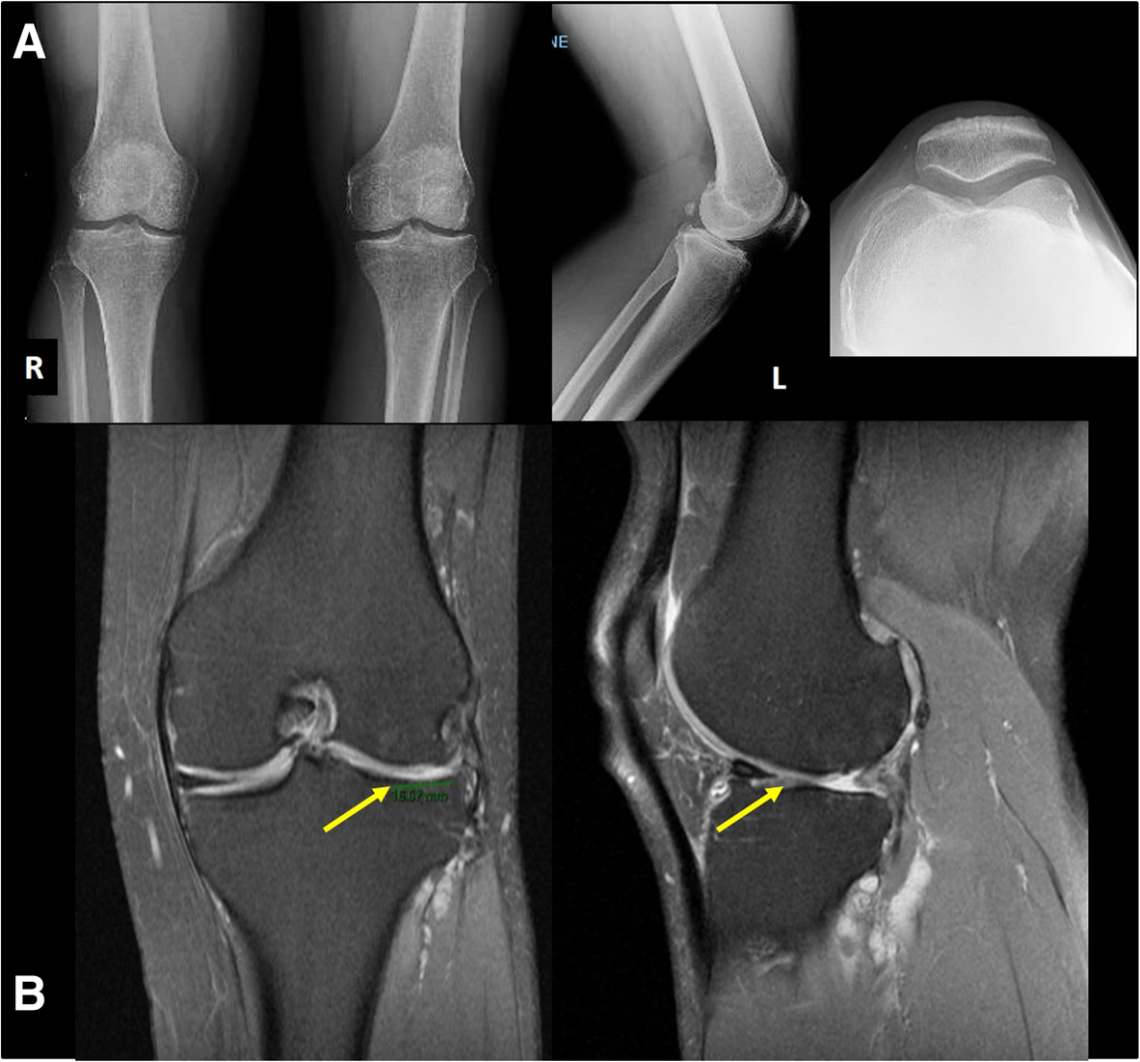
A 46-year-old man, with a grade IV cartilage injury of the lateral condyle and tibial plateau. **a** Preoperative plain radiographs. **b** Preoperative MRI showing a grade IV cartilage injury of the lateral condyle (30 × 20 mm) and grade III–IV cartilage injury of the tibial plateau (20 × 20 mm). Microfractures have been performed at the tibial plateau. The wider defect of the femoral condyle has been repaired with the Chondro-Gide® scaffold and BM-MSCs differentiated to chondrocytes

**Fig. 6:**
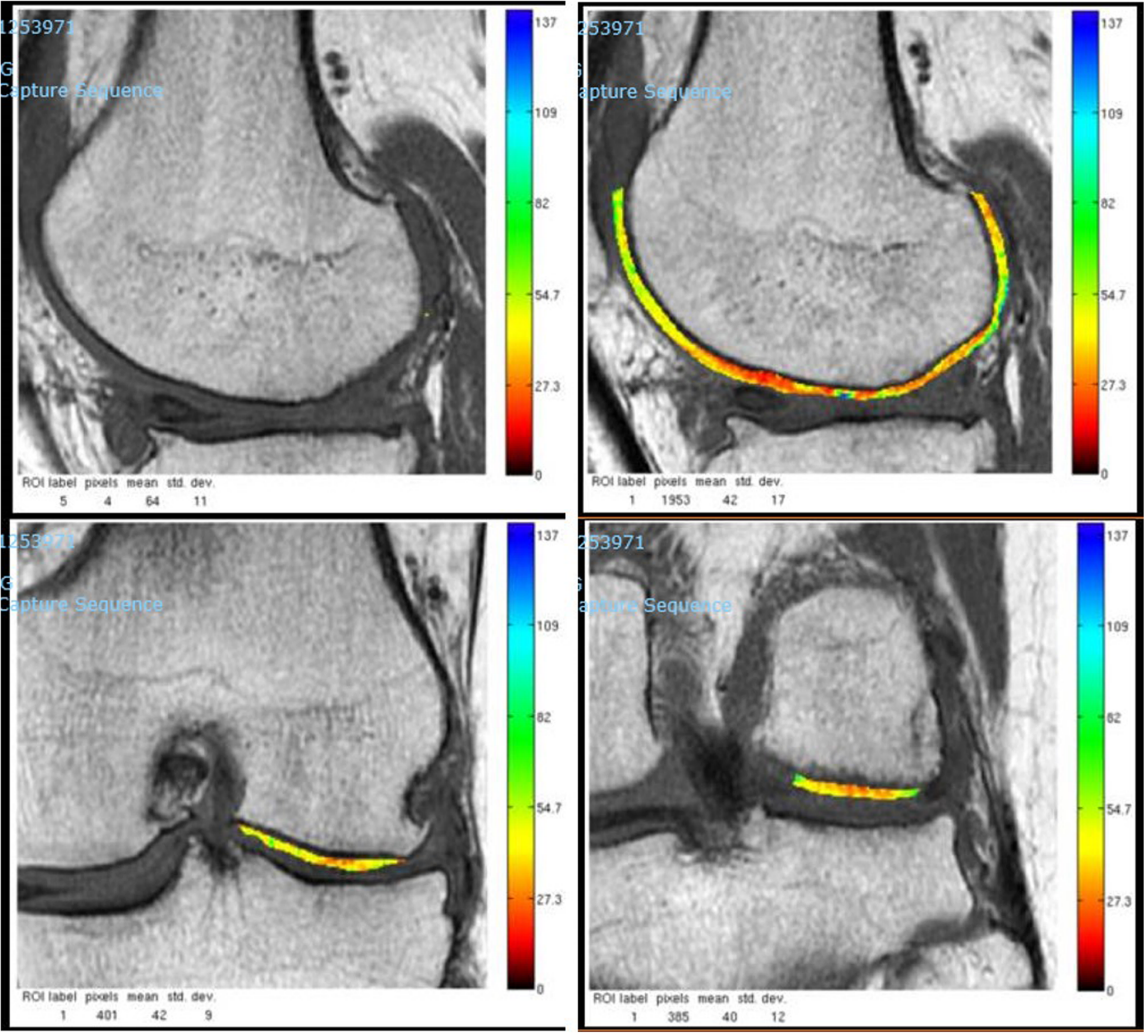
MRI of the same patient at 1 year follow-up. The T2 cartilage maps showed a full-thickness coverage of the defect with a heterogeneous and irregular tissue. The superficial layer of the tissue seems to have a higher content of water, while the layer close to the subchondral bone shows a structure more similar to normal cartilage. There is no bone edema in the area of the scaffold

**Fig. 7:**
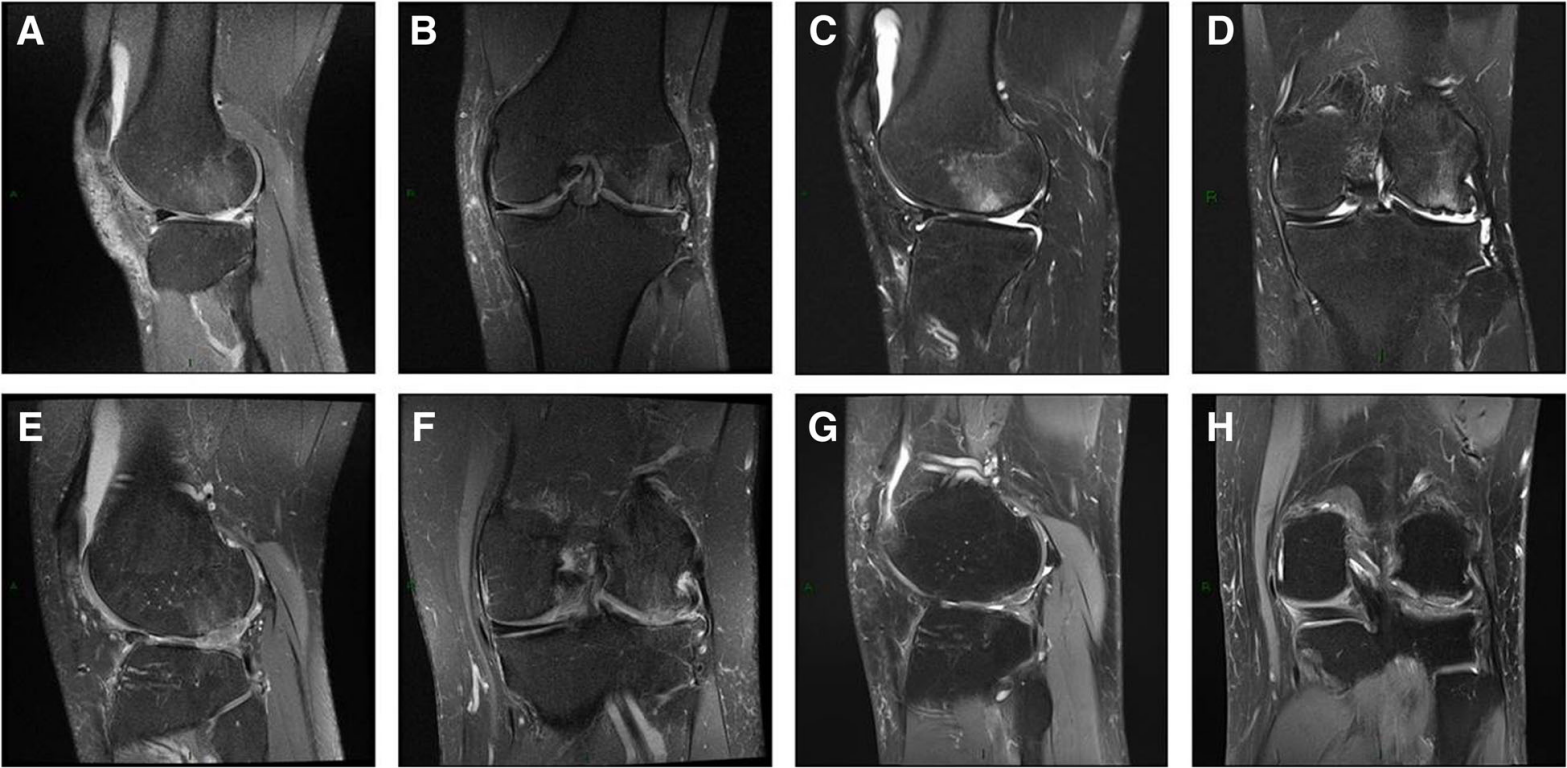
MRI post-surgery. Patient SPC: **a** sagittal fat-supressed proton density-weighted image 3 months after treatment. The graft tissue homogeneous filling the defect. Subchondral marrow edema. **b** Coronal fat-suppressed proton density-weighted image 3 months after treatment. Graft tissue homogeneous filling the defect. There is subchondral bone marrow edema, C: Sagittal fat-suppressed proton density-weighted image 53 months after treatment. The repair site is now filled with a congruent articular surface. Normal signal of the cartilage. Persistent bone marrow. **d** Coronal fat-suppressed proton density-weighted image 53 months after treatment. The repair site is now filled with a congruent articular surface. Persistent bone marrow edema and a focal irregularity in superficial layer are present. Patient CRC: **e** Sagittal fat-suppressed proton density-weighted image 6 months after the index procedure. The graft tissue inhomogeneous fills the defect with overgrowth of repair cartilage. Subtle subchondral marrow edema. **f** Coronal fat-suppressed proton density-weighted image 6 months after treatment. There is graft tissue in homogeneously filling the defect with overgrowth of the repaired cartilage. Subtle subchondral marrow edema. **g** Sagittal fat-suppressed proton density-weighted image 15 months after treatment. The repair site shows evidence of progression of tissue incorporation and a congruent articular surface. There is no bone marrow edema. **h** Coronal fat-suppressed proton density-weighted image 15 months after treatment. The repair site is now filled with incorporated tissue, and a congruent articular surface is evident. There is no bone marrow edema

At MRI at the final follow-up, the T2 cartilage maps showed a full-thickness coverage of the defect in all patients. At the site of the repair, the T2 mapping showed the presence of a heterogeneous tissue, more similar to the near healthy cartilage (Figs. 5, 6, and 7).

## Discussion

Cartilage injuries of the knee are a common major cause of pain and disability, and they are difficult to manage. Given their histological features and the lack of blood supply, full-thickness hyaline cartilage defects have limited healing potential and usually evolve to early osteoarthrosis [1]. Multiple therapeutic strategies have been used to manage cartilage injuries, including microfractures, scaffolds, regenerative therapies, and combination techniques [12]. However, the great variability of treatments, the relatively small study sizes, the short-term follow-up of most of the studies, and the lack of level I studies make it difficult to identify a gold standard procedure [13].

Great interest has been invested on cell-based therapies to improve cartilage healing, and autologous chondrocytes implantation has produced good clinical outcomes. Good clinical and functional outcomes have been confirmed by clinical trials at 10 years follow-up for large (> 4 cm^2^) full-thickness cartilage defects of the knee [2]*.* However, the donor site morbidity, the need of two-steps surgery, the costs of the procedures, the loss of the original properties of the graft secondary to fibrous cartilage-like tissue formation are the most frequent drawbacks of this procedure [14–16]. MSCs have recently been widely studied for regenerative medicine purposes. They are relatively simple to harvest and isolate, and they are able to self-renew and differentiate into specialized cells, making MSCs a promising treatment option for a variety of clinical conditions [17].

Currently, three different MSCs transplantation protocols exist. The first consists in the aspiration and concentration of MSCs [18]. Bone marrow (BM-MSCs) or adipose tissues (Ad-MSCs) are the most common sources of MSCs [6]. Many different devices are available which allow to harvest and concentrate MSCs, obtaining a mixed cell population, including also erythrocytes, leukocytes, and endothelial cells. After preparation, MSCs can be seeded on a scaffold or injected into the joint. These products do not imply substantial cell manipulation, making their use easier and less expensive, because all the treatment can be performed in a single procedure. However, the amount of MSCs is usually lower compared to the other protocols, although this does not imply that it is less effective [6], with several clinical and histological studies showing the formation of cartilage-like tissue, and good results at short-term follow-up [6, 19–21].

The second protocol consists into harvest, isolation, and expansion in vitro of MSCs, prior to implantation with a scaffold or by injection [13, 22]. The in vitro step allows to select a more homogeneous cell population. The number of cells administered to the patient is higher than the previous method, and it can be precisely determined. However, this procedure requires two steps, and it is usually more expensive. Few clinical studies have been published on knee and hip defects, showing promising results regarding pain and functional scores [23]. The authors recently published on 20 patients (29 hips) treated with hip arthroscopy for femoro-acetabular impingement (FAI) and focal cartilage injuries, or mild to moderate hip osteoarthrosis, followed by 3 intra-articular injections of expanded BM-MSCs (20 × 10^6^ BM-MSCs) from 4 to 6 postoperative weeks [24]. An improvement in VAS score and all functional scores in most of patients were reported at 2 years. No major complications were reported, but transient hip pain was present in 6 patients. Other authors reported promising results on focal cartilage defects and osteoarthritis of the knee [25, 26]. However, many concerns related to the extensive in vitro cell manipulation still exist. Furthermore, strict laws exist on the in vitro cell manipulation, making more complex their use in vivo for clinical trials or clinical purposes.

The last method consists in the isolation, expansion, and pre-differentiation in vitro of MSCs. MSCs are seeded onto a scaffold, cultivated, and pre-differentiated in vitro into chondrocytes for 2 to 4 weeks before implantation into the chondral defect. Pre-clinical studies showed promising results with the use of pre-cultivated MSCs. In animal studies, Zscharnach et al. [27] and Marquasset et al. [28] reported that pre-cultivated MSCs produced better repair tissue compared to concentrated MSCs, both at 6 weeks and 1 year. Histological analysis showed better cells distribution, matrix production, and a superior International Cartilage Repair Society (ICRS) score compared to controls. Furthermore, no degradation of the repair tissue was observed up to 12 months [28].

**Fig. 8:**
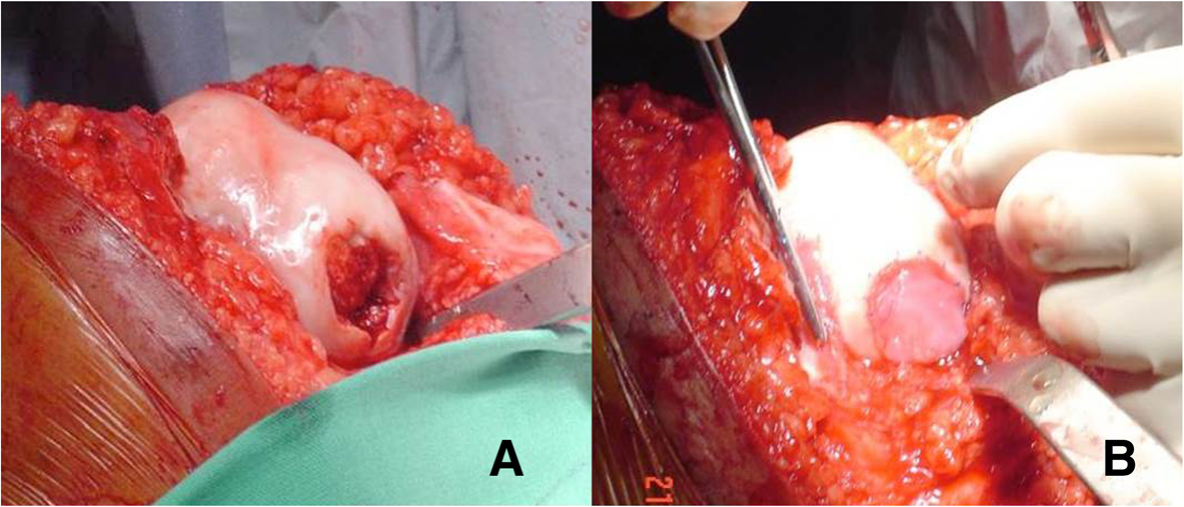
A 32-year-old patient with a grade IV cartilage injury of the lateral femoral condyle. **a** A large grade IV defect has been identified, and the cartilage defect has been prepared. **b** The Chondro-Gide® scaffold containing BM-MSCs differentiated to chondrocytes has been sutured onto the peripheral healthy cartilage with n.6.0 non-absorbable suture

Even though many authors investigated the differentiation potentials of MSCs in vitro, several limitations have been encountered during their use in vivo. Both BM-MSCs transplantation and non-pre-cultivated, or isolated and expanded MSCs transplantation, resulted in encouraging clinical outcomes, with the growth of hyaline-like cartilage based on imaging, arthroscopic, and histological examination [13]. However, there is still lack of evidence on the differentiation of MSCs into chondrocytes in clinical human studies [16]. Pre-differentiated to chondrocytes MSCs showed very encouraging result in animals, suggesting that they could be used in the treatment of osteochondral defects, but to our knowledge, no clinical studies have been published. To our knowledge, therefore, this is the first human clinical study on the treatment of focal full-thickness cartilage injury of the knee with using differentiated to cartilage BM-MSCs (Fig. 8).

Using pre-differentiated MSCs, we are able exploit many of the advantages of MSCs therapy [13]. First, harvesting of 40 mL bone marrow allowed to collect a number of MSCs sufficient to prevent expansion failure [29]. Furthermore, the stimulation of the chondrogenesis allows to increase the mechanical stability of the implant. This should improve the long-term outcome. We performed a knee arthrotomy to directly suture the Chondro-Gide scaffold to the surrounding healthy articular cartilage in patients who present large and/or multiple full-thickness cartilage defects, and in patients in whom previous arthroscopic management has failed. We are aware that in the present study patients were followed for a little less than 3 years. Nevertheless, many patients had resumed sporting activities (running, spinning, and long-distance running) without problems or pain, and others referred no pain and no swelling of the operated knee at the final follow-up. About 80% of the patients were very satisfied or satisfied with the surgery, and only one patient, who had undergone multiple knee procedures, reporting to be unsatisfied. The need of a two-steps procedure, the long time between the harvesting of BM-MSCs, and the final implantation of the scaffold (4 weeks), the higher risk of contamination compared to the one-step procedure, and the higher costs, are the most important limits of this procedure.

We are aware of the limitations of our study. The first is the small size of our cohort, 19 knees in 15 patients. The middle-term follow-up did not allow us to draw final conclusions on longer-term outcomes of these procedures. However, most of the studies published in literature have a follow-up shorter than 3 years. We point out that this is a preliminary study, and we aim to continue to follow them to publish a study with longer-follow-up. Also, there was no control group, but to our knowledge, no published studies compared MSCs-therapy to controls or other techniques. Many patients had undergone previous knee surgeries, including microfractures, partial meniscectomy, ACL reconstruction, and medial patellofemoral ligament reconstruction. Our clinic is a tertiary referral center for sports traumatology, accounting for the fact that some patients had been treated previously in other hospitals and were subsequently referred to us after failure of a variety of treatments, including previous surgery.

## Conclusion

Full-thickness cartilage injuries of the knee are major causes of pain and disability. Despite all the recent efforts, no procedure can guarantee success, and the published long-term results of many procedures are not satisfactory. MSCs showed great potentials in the treatment of this complex pathology, even though the complexity of MSCs metabolism and their therapeutic effects does not allow to draw definitive conclusions. We report promising results using BM-MSCs differentiated to chondrocytes and collagen type I/III scaffold for treatment of full-thickness chondral injuries of the knee. However, further studies involving more patients, and with longer follow-up, are needed to evaluate the effectiveness of this treatment modality and its long-term results.

## Data Availability

All data generated or analyzed during this study are included in this published article. The datasets used and/or analyzed during the current study are also available from the corresponding author on reasonable request.

## Abbreviations

MSCs: Mesenchymal stem cells
BM-MSCs: Bone marrow-MSCs
AD-MSCs: Adipose-derived-MSCs
MPFL: Medial patella-femoral ligament
ACL: Anterior cruciate ligament
